# Salvage total hip arthroplasty after internal fixation compared with acute total hip arthroplasty for fracture: a cohort study based on 32,960 cases from the Dutch Arthroplasty Register

**DOI:** 10.2340/17453674.2026.46046

**Published:** 2026-06-12

**Authors:** Laura VAN MARLE, Rinne M PETERS, Liza N VAN STEENBERGEN, Ruurd L JAARSMA, Job N DOORNBERG, Wierd P ZIJLSTRA

**Affiliations:** 1 Department of Orthopedic Surgery, Frisius Medical Center Leeuwarden; 2 Department of Orthopedic Surgery, University Medical Center Groningen, The Netherlands; 3 Department of Orthopedic Surgery, Flinders Medical Center, Adelaide, Australia; 4 Dutch Arthroplasty Register (LROI), ‘s Hertogenbosch, The Netherlands

## Abstract

**Background and purpose:**

Total hip arthroplasty (THA) is used as treatment of acute hip fracture and as a salvage procedure after failed internal fixation (IF). We aimed to assess revision rates of salvage-THA after IF of hip fracture compared with acute fracture-related THA.

**Methods:**

Using the Dutch Arthroplasty Register, 32,960 procedures with fracture-related THAs of the proximal femur between 2007 and 2023 were selected. We performed competing risk survival analysis and adjusted multivariable Cox regression.

**Results:**

12,486 patients received salvage THA after failed IF, 744 (6.0%) needed revision THA; 18,450 patients received acute THA for fracture, 885 (4.8%) needed revision THA. Cumulative rates of revision THA after salvage THA at 1 and 5 years were 2.7% (95% confidence interval [CI] 2.4–3.1) and 4.5% (CI 4.1–4.9), respectively, comparable to 2.5% (CI 2.3–2.8) and 4.3% (CI 4.0–4.6), for revision THA after acute THA. Revision THA of salvage THA patients were more often younger, male, had higher BMI, lower ASA class, and were smokers. Posterolateral approach, reversed-hybrid fixation, and small femoral heads were more common, compared with revision after acute THA for fracture patients. In both groups, most common reasons for revision THA were infection, aseptic loosening, and dislocation. In the salvage THA, adjusted hazard ratios (HR) showed increased risk of revision THA for ASA class III–IV (HR 1.6), high BMI (BMI > 25, HR 1.6–2.1), reversed hybrid fixation (HR 1.3), and 22–28 mm femoral head size (HR 1.3). Cumulative second revision rates of salvage THA after IF at 1 and 5 years were 14.1% (CI 11.6–17.2) and 22.3% (CI 19.0–26.2), similar to the acute THA for fracture group.

**Conclusion:**

Revision rates after salvage THA following IF were comparable to those after acute THA for fracture. Hence, previous internal fixation does not seem disadvantageous. However, both groups differed clearly in case mix.

The number of patients presenting with proximal femoral fractures (PFF) is increasing and contributing significantly to the global healthcare burden, partially due to the aging population [1]. Surgical treatment depends on patient characteristics and fracture pattern. While internal fixation (IF) remains a common choice of treatment, it may result in failure due to non-union, mechanical failure, avascular necrosis, posttraumatic osteoarthritis, and implant-related infection, for which conversion to a total hip arthroplasty (THA) might be indicated as a salvage procedure [2]. An increasing proportion of patients are treated with primary arthroplasty (either hemiarthroplasty or total hip arthroplasty).

Both patient- and surgery-related factors contribute to an increased risk of unfavorable outcomes following salvage THA after previous IF [3]. Patients undergoing salvage THA after failed IF are more at risk of perioperative complications [5]. It is therefore clinically relevant whether the outcome of (a delayed) THA after IF differs from THA for an acute fracture.

The primary aim of our study was to assess the risk of revision of salvage THA after failed IF compared with acute fracture-related THA. Second, we aimed to determine common reasons for revision after salvage THA and acute fracture-related THA and identify risk factors by investigating whether there is an association between patient characteristics or surgically modifiable factors and the risk of revision THA.

## Methods

### Study design

In this nationwide population-based observational registry study, data from the Dutch Arthroplasty Register (LROI) was retrieved. The LROI was initiated by the Dutch Orthopaedic Association in 2007. The level of completeness of the LROI is excellent: 98% and 99% of all primary THA and revision hip procedures are registered, respectively [4]. The STROBE and ICMJE guidelines were taken into account [6,7].

### Patients and outcome

All primary THA for acute proximal femoral fracture (acute THA), and all salvage THA after previous IF (salvage THA) on the ipsilateral hip between 2007 and 2023 were retrieved from the register (n = 32,960); bilateral procedures were included. Exclusion criteria for the salvage THA group were: (i) metal-on-metal (MoM) articulation and (ii) diagnosis other than late posttraumatic, osteonecrosis, osteoarthritis, or fracture. Late posttraumatic is defined as > 5 days after trauma on the LROI Hip Primary registration form. For the acute THA group, the diagnosis was fracture. Other diagnoses were excluded. Risk of first revision after the primary THA was defined as primary outcome variable. Secondary outcomes were second revision rate, as well as reason for revision, and patient and procedure characteristics. Revision arthroplasty was defined by the LROI as every modification of one or more components of the prosthesis. Multiple reasons for revision can be registered in the LROI at the time of the revision, e.g., a revision might be performed for septic loosening and femoral loosening and infection can all be registered simultaneously. For analytical purposes, these were hierarchized based on clinical relevance [8]. Loosening of the acetabular or femoral component without infection was combined into aseptic loosening. Femoral head and construct were categorized as dual mobility cup, 22–28 mm, 32 mm and ≥ 36 mm femoral head.

### Statistics

Patient and procedure characteristics were summarized. Survival time was defined as the time from primary THA to first revision procedure, death of the patient or end of the follow-up period (January 1, 2024). Cumulative crude incidence of revision was calculated using competing risk analysis; death was considered as competing risk. Competing risk analysis provides more insight in the risk of surgical revision by taking both implant failure and mortality into account, as opposed to Kaplan–Meier analysis [9,10]. The crude cumulative incidence of revision was determined at 1, 3, 5, 10, and 15 years after primary THA. For second revisions, this was calculated at 1, 3, 5, and 8 years. Multivariable Cox regression analysis was performed to account for differences in case-mix; age, sex, American Society of Anesthesiologists Physical Status classification (ASA) and body mass index (BMI), fixation, surgical approach (first revision only), and femoral head size (first revision only) were entered as confounders. Confounders were selected based on available variables and statistically significant crude hazard ratios and expert clinical judgment regarding relevance in context (Supplementary Figure 1). P values < 0.05 were considered significant. SPSS version 28 (IBM Corp, Armonk, NY, USA) was used for all statistical analysis.

Given the exploratory nature of this registry-based study and the variation in findings, we performed the following post-hoc analyses to uncover patterns or other influential factors.

Additional analyses were performed to examine the association of period of primary THA (as a proxy for time trends in techniques and prosthesis components) and risk of revision. Period of primary THA was categorized (2007–2013, 2014–2018, 2019–2023) and included in a uni- and multivariable Cox-regression analysis.

Furthermore, risk factors for revision according to reason (infection, dislocation, aseptic loosening, and periprosthetic fracture) were assessed using multivariable Cox regression analyses.

### Ethics, registration, data sharing plan, funding, and disclosures

The study was approved by the scientific advisory board of the LROI and exempted for ethical approval according to the Dutch Medical Research with Human Subjects Act (WMO). Data was registered confidentially with patient consent, and all data is processed anonymously. All data was received completely de-identified. The LROI uses the opt-out system to require informed consent from patients during usual care. Bound to privacy regulations, sharing of data is not permitted. This study was funded by a grant from the Dutch Arthroplasty Register (grant number: LROI RG 2024-001). The authors declared no conflicts of interest. Complete disclosure of interest forms according to ICMJE are available on the article page, doi: 10.2340/17453674.2026.46046

## Results

**Figure 1:**
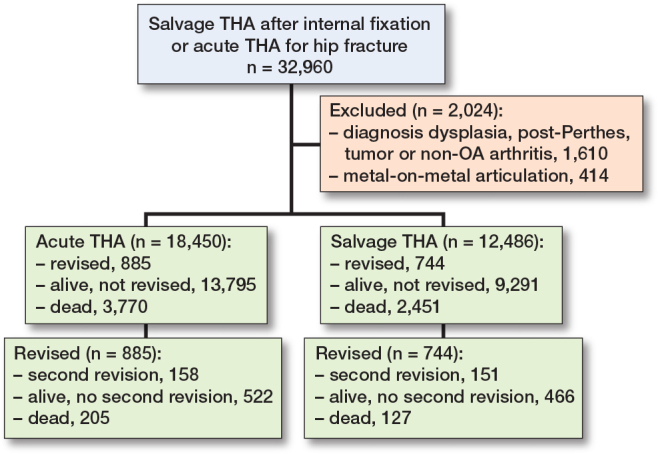
Flowchart of included total hip arthroplasty (THA) procedures and outcome.

12,486 salvage THAs after previous IF were identified with a mean survival of 6.1 years (SD 4.2), and 18,450 acute THAs, with a mean survival of 5.1 years (SD 4.1). A first revision was performed in 6.0% (744/12,486), a second revision in 20% (151/744) in the salvage THA cohort. In the acute THA cohort, revision rates were 4.8% (885/18,450) and 18% (158/885) respectively (Figure 1).

### Case mix

Substantial differences in case mix between salvage THA and acute THA for fracture were observed. Patients in the salvage-THA cohort were generally younger, more often male, had lower ASA class, higher BMI, and higher smoking prevalence. Diagnoses reported for salvage THA were mostly “late posttraumatic” (55%), and to a lesser extent osteoarthritis (29%) or osteonecrosis (16%). Posterolateral approach, reversed hybrid fixation, and slightly more small femoral heads at primary procedure were more frequently encountered in the salvage-THA group. The use of a dual mobility (DM) construct was similar (Supplementary Table 1).

### Crude incidence of revision

**Figure 2:**
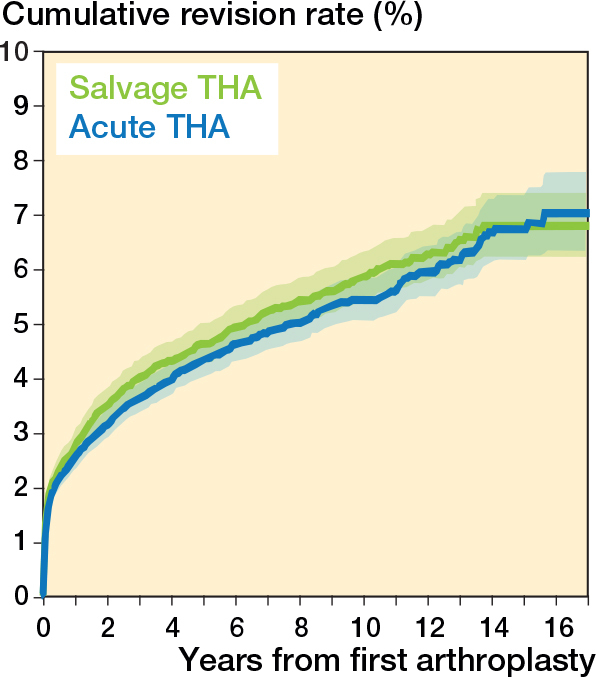
Cumulative incidence of first revision after salvage THA and acute THA for fracture.

The crude cumulative incidences of revision between salvage THA and acute THA were comparable (Table 1, Figure 2). Rates at 1 and 10 years were respectively 2.7% (95% confidence interval [CI] 2.4–3.1) and 5.7% (CI 5.3–6.2) for salvage THA, and 2.5% (CI 2.3–2.8), and 5.4% (CI 5.0–5.8) for acute THA after fracture. Mortality at 5 and 10 years was respectively 13% (CI 12.5–13.8) and 29% (CI 28.1–30.3) in the salvage-THA cohort, and 16% (CI 15.6–16.8) and 34% (CI 32.6–34.7) in the acute-THA cohort. Infection was the most common reason for revision in salvage-THA cohort, and dislocation was the most common reason in the acute-THA cohort (Table 2).

**Table 1:** Crude cumulative incidence of first revision of salvage THA after internal fixation or acute THA

Follow-up	At risk, n	Crude cumulative incidence % (CI)
Salvage THA (n = 12,486)		
1 year	11,044	2.7 (2.4–3.0)
3 years	8,757	3.9 (3.6–4.3)
5 years	6,709	4.5 (4.1–4.9)
10 years	2,503	5.7 (5.3–6.2)
15 years	208	6.7 (6.1–7.3)
Acute THA (n = 18,450)		
1 year	15,625	2.5 (2.3–2.8)
3 years	11,408	3.6 (3.3–3.9)
5 years	8,024	4.3 (4.0–4.6)
10 years	2,529	5.4 (5.0–5.8)
15 years	418	6.8 (6.2–7.5)

CI = 95% confidence interval; THA = total hip arthroplasty.

**Table 2:** Reasons for revision of salvage THA after internal fixation and acute THA, hierarchized. Values are count (%)

	Salvage THA	Acute THA
First revision **^a^**	744	885
Infection	209 (28)	179 (20)
Aseptic loosening	197 (26)	205 (23)
Periprosthetic fracture	87 (12)	138 (16)
Dislocation	146 (20)	262 (30)
Other	89 (12)	88 (9.9)
Second revision **^b^**	151	158
Infection	76 (50)	77 (49)
Aseptic loosening	20 (13)	21 (13)
Periprosthetic fracture	5 (3.3)	8 (5.1)
Dislocation	18 (12)	28 (18)
Other	29 (19)	21 (13)

a Missing: Salvage THA n = 16 and acute THA n = 13.

b Missing: Salvage THA n = 3 and acute THA n = 3.

### Risk factors within the salvage-THA cohort

Patients aged under 60 years had a higher risk of revision (hazard ratio [HR] 1.3, CI 1.0–1.6). Moreover, higher ASA classification (III–IV; HR 1.4, CI 1.1–1.8), obesity (BMI 30–40; HR 1.5, CI 1.2–1.9), and morbid obesity (BMI > 40; HR 2.6, CI 1.2–5.6), were associated with an increased risk of revision. Anterior surgical approach (HR 0.7, CI 0.5–0.9) or straight lateral (HR 0.8, CI 0.6–0.9) were associated with reduced revision risk. Prosthesis-related factors associated with an increased revision risk were reversed hybrid fixation (HR 1.3, CI 1.0–1.8) and 22–28 mm femoral head (HR 1.3, CI 1.0–1.6) (Table 3).

**Table 3:** Multivariable Cox regression survival analysis of revision risk by patient and procedure factors during salvage THA after internal fixation

Item	First revision HR (CI) **^a^**	Second revision HR (CI) **^b^**
Age, years		
< 60	1.3 (1.0–1.6)	1.0 (0.6–1.7)
60–75	1.0 (0.8–1.3)	1.0 (0.6–1.6)
> 75	1.0 (ref.)	1.0 (ref.)
Sex		
Male	1.0 (ref.)	1.0 (ref.)
Female	0.8 (0.7–1.0)	0.8 (0.7–1.0) **^c^**
ASA class		
I	1.0 (ref.)	1.0 (ref.)
II	1.2 (0.9–1.4)	1.1 (0.7–1.8)
III–IV	1.6 (1.2–1.9) **^d^**	1.7 (1.0–3.0)
Body mass index (BMI)		
< 18.5	0.7 (0.4–1.5)	2.9 (1.0–8.4)
18.5–25	1.0 (ref.)	1.0 (ref.)
25–30	1.0 (0.8–1.3)	1.8 (1.2–2.7) **^c^**
30–40	1.6 (1.2–2.0) **^d^**	2.7 (1.7–4.2) **^d^**
> 40	2.1 (0.8–5.7)	
Approach		
Anterior	0.7 (0.5–1.0) **^c^**	
Anterolateral	1.2 (0.8–1.6)	
Posterolateral	1.0 (ref.)	
Straight lateral	0.8 (0.6–1.0) **^c^**	
Fixation		
Cemented	1.0 (ref.)	1.0 (ref.)
Cementless	1.2 (1.0–1.4)	1.0 (0.7–1.5)
Hybrid: femur cemented	1.3 (0.9–1.7)	0.6 (0.3–1.2)
Reverse hybrid: cup cemented	1.3 (1.0–1.8) **^c^**	0.8 (0.4–1.4)
Construct and femoral head size		
Dual mobility cup	1.2 (0.9–1.6)	
22–28 mm	1.3 (1.0–1.6) **^c^**	
32 mm	1.0 (ref.)	
≥ 36 mm	1.1 (0.9–1.4)	
Diagnosis		
Late posttraumatic	1.0 (ref.)	
Osteoarthritis	0.7 (0.6–0.9) **^d^**	
Osteonecrosis	0.8 (0.6–1.0) **^c^**	

For abbreviations, see Table 1 and ASA = American Society of Anesthesiologists Physical Status classification.

Hazard ratios (HR) with 95% confidence interval (CI) adjusted for:

a age, sex, ASA class, BMI, fixation, approach, femoral head size;

b age, sex, ASA class, BMI, fixation.

c P < 0.05.

d P < 0.001.

The time of salvage THA was not associated with substantial change in HR (data not shown).

Associations were assessed for the specific reasons for revision. Risk of revision for infection was associated with younger age (HR 1.9, CI 1.2–2.9), high ASA (HR 2.2, CI 1.3–3.7), dual mobility cup (HR 1.7, CI 1.0–3.1), and higher BMI (BMI 25–30; HR 1.7, CI 1.2–2.4, BMI 30–40; HR 2.6, CI 1.7–3.9). Risk of revision for dislocation was associated with older age (> 75 years; HR 0.6, CI 0.4–1.0), female sex (HR 0.6, CI 0.4–0.9), straight lateral approach (HR 0.5, CI 0.3–0.8), dual mobility cup (HR 0.3, CI 0.1–0.7), and small femoral head size (22–28 mm; HR 1.6, CI 1.0–2.4). Risk of revision for aseptic loosening was associated with reversed hybrid fixation (HR 2.5, CI 1.5–4.2). Risk of revision for periprosthetic fracture was associated with higher ASA class (ASA II; HR 3.4, CI 1.3–8.8, ASA III–IV; HR 5.4 CI 1.9–15.1), cementless (HR 8.2, CI 3.3–20.0) or hybrid fixation (HR 4.1, CI 1.3–13.0), and larger femoral head size (≥ 36 mm; HR 2.1, CI 1.2–3.7) (data not shown).

### Second revision of THA

**Figure 3:**
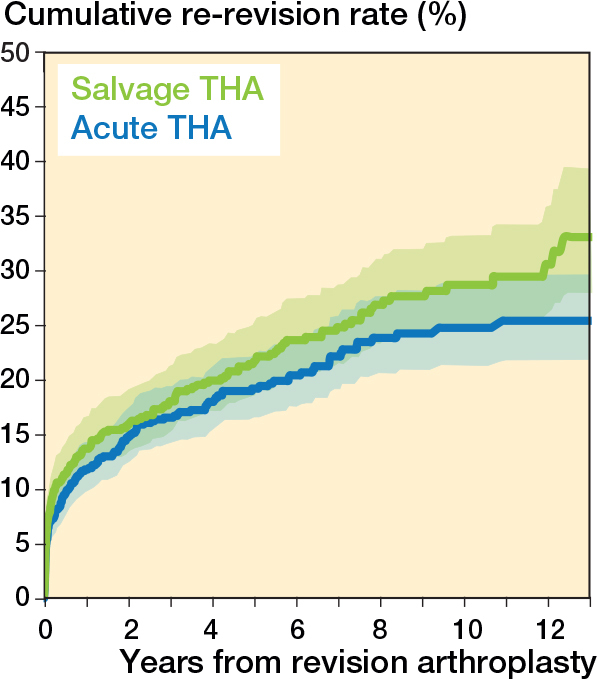
Cumulative incidence of second revision after salvage THA and acute THA for fracture from first revision.

The crude cumulative incidence of second revision was comparable between the salvage-THA cohort and the acute-THA cohort: 14% (CI 11.6–17.2) at 1 year, 22% (CI 19.0–26.2) at 5 years, and 28% (25.4–33.2) at 8 years in the salvage-THA cohort, as opposed to 11% (9.3–13.8), 19% (16.5–22.5), and 25% (20.9–28.3) in the acute-THA cohort (Figure 3, Table 4). Most common reasons for revision were similar for both salvage THA and acute THA (see Table 2). Higher risk of second revision in male sex (HR 1.4, CI 1.0–2.0) was found, as well as in those with higher BMI (BMI 25–30; HR 1.8, CI 1.2–2.7, BMI >30; HR 2.7, CI 1.7-4.2) (Table 3).  

**Table 4:** Crude cumulative incidence of second revision of salvage THA after internal fixation or acute THA

Follow-up	At risk, n	Crude cumulative incidence % (CI)
Salvage THA (n = 744)		
1 year	453	14.1 (11.6–17.2)
3 years	323	18.9 (16.0–22.5)
5 years	225	22.3 (19.0–26.2)
8 years	110	27.7 (23.8–32.2)
Acute THA (n = 885)		
1 year	602	11.3 (9.3–13.8)
3 years	380	16.4 (13.9–19.3)
5 years	235	19.2 (16.5–22.5)
8 years	107	24.3 (20.9–28.3)

For abbreviataions, see Table 1.

## Discussion

We evaluated first and second revision rates following salvage THA of hip fractures in the Netherlands. We found a 5- and 10-year revision rate of 4.5% and 5.7%, which was comparable to the revision rates after acute THA for fracture. There were, however, notable differences in patient and procedure characteristics between patients receiving salvage THA, and patients receiving acute THA for fracture. The most frequent reasons for revision were infection, aseptic loosening, and dislocation. Patient-related factors associated with an increased risk of first revision after salvage THA were high ASA class and high BMI. Procedure-related factors resulting in an increased risk of first revision were small (22–28 mm) femoral head size and reversed hybrid fixation, whereas anterior and straight lateral approach were associated with a decreased revision risk. The cumulative incidence of second revision was 14% at 1 year, and 28% at 8 years, most commonly due to infection and dislocation. Male sex and a high BMI were associated with a significantly increased risk of second revision.

The cumulative incidence of revision of salvage THA in our study was comparable to the incidence in the acute fracture-related THA cohort, although patient and procedure characteristics differed significantly, as was discovered previously [11]. According to the LROI, the risk for revision following primary THA in patients with osteoarthritis after 5 and 10 years is around 3% and 5% [4]. The increased revision rates in the posttraumatic cohort uncovered in our study align with previous (registry-based) reports that describe increased failure rates in the general posttraumatic cohort, despite the inability to differentiate the different types of proximal femoral fractures in all studies including ours [3,12-16]. The mortality rates in the salvage-THA cohort appeared to be faintly lower than in the acute-THA cohort.

Higher BMI was the only factor associated with increased rates of both first and second revision for salvage THA. When zooming in on specific reasons for revision, hazard ratio increases for infection, suggesting BMI is playing a major role in the pathway of failure of THA after IF due to infection. Infection being the main reason for second revision, this consistent pattern suggests that patient-related factors play an important role throughout the revision pathway. Previously reported common reasons for revision after salvage THA after IF, i.e., infection, dislocation, and femoral loosening, are supported by our findings [17,18]. Multiple studies showed an association between periprosthetic infection and high ASA, high BMI, long operative time, diabetes, anemia, and urinary tract infection, which are common characteristics in the older, frail population [19].

For periprosthetic fractures, Lamb et al. (2025) reported a higher incidence than previously estimated due to the lack of registration of surgical procedures other than revision surgery. Both stem design and fixation method proved to influence revision rates, as well as fracture pattern [5,20]. Similarly, cementless or hybrid fixation was associated with increased revision rates of salvage THA, as well as large femoral head size (≥ 36 mm). The stability and anatomy of the femur can be affected by the previous internal fixation and/or removal of the metalwork, attributing to the risk of failure by femoral loosening [21]. These findings highlight the nuance of balancing implant choice, cementation technique, and other factors, rather than a cemented vs cementless comparison.

The conversion of failed IF to THA has been reported to carry higher risks of instability and periprosthetic fractures compared with primary THA [22]. Our results add to previous findings that support the use of a dual-mobility construct and avoidance of small femoral heads in fracture-related THA to reduce the rate of revision due to dislocation [23,24].

### Limitations

In frailer patients, extensive revision procedures might not be pursued after shared decision-making, due to either increased surgical risk or limited expected benefit. The association of higher ASA class with periprosthetic fracture and infection, and older age with dislocation, shows revisions are performed in the presumed frailer individuals.

Tetsunaga et al. found an increased risk of complications after salvage THA among previous trochanteric fractures [25]. In cases of failed previous internal fixation, especially if a dynamic hip screw or intramedullary nail was used, most surgeons will probably use the same approach for extracting the osteosynthesis and implanting the THA, e.g., posterolateral (PL) and straight lateral (SL). Additionally, fracture fixation is commonly performed using a lateral surgical approach. This may explain why the anterior approach, while currently used in 50% of cases in the Netherlands for primary osteoarthritis, is only sparsely used in this cohort, and the majority had a posterolateral or straight lateral approach. Whether the association of surgical approach with risk of revision is based on the above, or on a reduced risk of dislocation compared with posterolateral, cannot be fully answered based on our data. The register does not capture whether the same surgical approach and incision were used for IF as for conversion to THA.

As observational data was used no conclusions on causality can be drawn. Due to missing data, several cases had to be excluded. Lastly, the register lacked detailed information on fracture classification or initial fixation methods, patient-reported outcome measures (PROMs) after hip fracture fixation, and patient or surgeon preference for revision; LROI does not register hip fracture surgery with internal fixation. Strengths of this study are the use of a large dataset based on real-world registry data. Furthermore, this study helps in clinical decision-making concerning the choice for a hip prosthesis or internal fixation in patients with hip fractures.

Understanding the elevated risk of revision after salvage THA is crucial for clinical decision-making. As illustrated by the different baseline characteristics in our material, younger and healthier patients are often initially treated with IF whereas older or more comorbid patients are preferentially treated with some type of arthroplasty. Despite this selection bias, we found no difference in revision rate when comparing acute THA and salvage THA. Infection, aseptic loosening, and dislocation are the most common reasons for failure in fracture-related THA, especially in patients with high ASA and BMI. Failure may result from a combination of patient factors such as osteoporosis, obesity, diabetes, and general frailty [26]. This information should be used in the preoperative shared decision-making process between the surgeon and the patient. Knowledge of modifiable surgical risk factors, such as implant selection and approach, may assist surgeons in optimizing outcomes. Small femoral heads should be avoided to reduce the risk of dislocation [27].

Infection was the main reason for first and second revision in our cohort, indicating patients’ frailty and the added risk of multiple surgeries. In cases of failed fixation especially, it is paramount to rule out infection prior to salvage THA and take appropriate measures to avoid infection [19,28].

### Conclusion

Salvage THA after internal fixation was associated with a first revision risk of 5% at 5 years’ follow-up, which was comparable to acute THA for fracture (4%). Additionally, a substantial risk of second revision at 5 years was found, 22% after salvage THA compared with 19% after acute THA. The most frequent reasons for revision were infection, aseptic loosening, and dislocation. Patient factors associated with increased first revision risk were high ASA and high BMI at primary procedure, and high BMI was also associated with increased second revision risk, especially for infection.

*In perspective,* these findings suggest that salvage THA can achieve revision rates comparable to acute THA despite being performed in a more complex and frail patient population. Preoperative risk assessment and optimization of modifiable factors are essential to improve outcomes and for shared decision-making.

### Supplementary data

Supplementary Table 1 and supplementary Figure 1 are available as supplementary data on the article page, doi: 10.2340/17453674.2026.46046

## Supplementary Material



## Notes

LvM contributed to gathering data, analyzing data, initial draft, final draft. RMP, WPZ, and LNvS contributed to study design and study protocol, gathering data, initial draft, and final draft. JND contributed to study design and study protocol, initial draft, and final draft. RLJ contributed to initial draft and final draft.

Handling co-editors: Cecilia Rogmark and J Ranstam

*Acta* thanks Jan-Erik Gjertsen and Henrik Palmrs for help with peer review of this manuscript.
