# Supplementary material for: Salvage total hip arthroplasty after internal fixation compared with acute total hip arthroplasty for fracture: a cohort study based on 32,960 cases from the Dutch Arthroplasty Register
Source: Acta Orthop. 2026 Jun 12;97:388–94. doi: 10.2340/17453674.2026.46046 (PMC13261751; doi:10.2340/17453674.2026.46046)
Supplement: Supplementary file 1 [file ActaO-97-46046-s1.pdf]

Supplementary data

Supplementary Table 1. Patient and procedure characteristics of salvage THA after previous internal fixation and acute THA in the Netherlands between 2007 and 2023 (n = 32,960)

	Salvage THA (n = 12,486)	Acute THA (n = 18,450)
Age, years	n = 10,448	n = 18,434
< 60	3,079 (25)	2,224 (12)
60–75	5,671 (46)	11,158 (61)
> 75	3,698 (30)	5,052 (27)
Sex	n = 12,475	n = 18,431
Female	7,450 (60)	12,422 (67)
Male	5,025 (40)	6,009 (33)
ASA class	n = 12,351	n = 18,092
I	2,193 (15)	2,633 (15)
II	7,084 (58)	10,482 (58)
III–IV	3,074 (25)	4,977 (28)
Body mass index [a]	n = 8,958	n = 13,864
< 18.5	277 (3.1)	471 (3.4)
18.5–25	4,004 (45)	7,219 (52)
25–30	3,297 (37)	4,701 (34)
30–40	1,333 (15)	1,411 (10)
> 40	47 (0.5)	62 (0.4)
Smoking [a]	n = 8,765	n = 14,083
Yes	1,502 (17)	1,975 (14)
No	7,263 (83)	12,108 (86)
Approach	n = 12,330	n = 18,144
Anterior	1,353 (11)	3,579 (20)
Anterolateral	625 (5.1)	1,052 (5.8)
Posterolateral	8,243 (67)	10,801 (60)
Straight lateral	2,109 (17)	2,712 (15)
Articulation	n =12,486	n = 18,450
Ceramic-on-ceramic	600 (4.8)	1,031 (5.6)
Ceramic-on-PE	6,721 (54)	10,516 (57)
Metal-on-PE	4,174 (33)	5,975 (32)
Oxidized zirconium-on-PE	991 (7.9)	928 (5.0)
Fixation	n = 12,396	n = 18,252
Cemented	4,440 (36)	6,400 (35)

Cementless	5,897 (48)	9,031 (50)
Hybrid: femur cemented	1,131 (9.1)	2,209 (12)
Reversed hybrid: cup cemented	928 (7.5)	612 (3.4)
Construct and femoral head size	n = 12,357	n = 18,200
Dual mobility cup	1,734 (14)	2,553 (14)
22–28 mm	2,276 (18)	3,098 (17)
32 mm	6,119 (50)	8,649 (48)
≥ 36 mm	2,228 (18)	3,900 (21)
Period	n = 12,486	n = 18,450
2007–2013	3,757 (30)	4,080 (22)
2014–2018	4,368 (35)	6,063 (33)
2019–2023	4,361 (35)	8,307 (45)
Diagnosis [b]	n = 12,486	
Late posttraumatic	6,845 (55)	
Osteoarthritis	3,676 (29)	
Osteonecrosis	1,965 (16)	

[a] *Registered since 2014.* [b] Solely for salvage-THA group.

Numbers might not add up due to missing data.

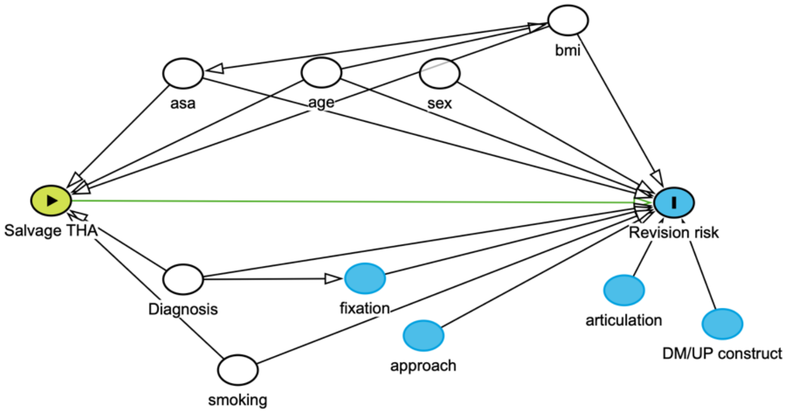

Supplementary Figure. 1 Directed acyclic graph.
